# Exploration of anti‐leukemic effect of soft coral‐derived 13‐acetoxysarcocrassolide: Induction of apoptosis via oxidative stress as a potent inhibitor of heat shock protein 90 and topoisomerase II

**DOI:** 10.1002/kjm2.12678

**Published:** 2023-04-13

**Authors:** Hsien‐Kuo Chin, Mei‐Chin Lu, Kai‐Cheng Hsu, Mohamed El‐Shazly, Tsen‐Ni Tsai, Tzu‐Yung Lin, Shou‐Ping Shih, Tony Eight Lin, Zhi‐Hong Wen, Yu‐Chen S. H. Yang, Yi‐Chang Liu

**Affiliations:** ^1^ Department of Marine Biotechnology and Resources National Sun Yat‐Sen University Kaohsiung Taiwan; ^2^ Division of Cardiovascular Surgery, Department of Surgery Kaohsiung Armed Forces General Hospital Kaohsiung Taiwan; ^3^ Graduate Institute of Marine Biology National Dong Hwa University Hualien Taiwan; ^4^ National Museum of Marine Biology and Aquarium Pingtung Taiwan; ^5^ Graduate Institute of Cancer Biology and Drug Discovery, College of Medical Science and Technology Taipei Medical University Taipei Taiwan; ^6^ Master Program for Cancer Molecular Biology and Drug Discovery, College of Medical Science and Technology Taipei Medical University Taipei Taiwan; ^7^ Ph.D. Program for Cancer Molecular Biology and Drug Discovery, College of Medical Science and Technology Taipei Medical University Taipei Taiwan; ^8^ TMU Research Center of Drug Discovery Taipei Medical University Taipei Taiwan; ^9^ Department of Pharmacognosy, Faculty of Pharmacy Ain‐Shams University Cairo Egypt; ^10^ Division of Hematology‐Oncology, Department of Internal Medicine Kaohsiung Medical University Hospital Kaohsiung Taiwan; ^11^ Department and Graduate Institute of Aquaculture National Kaohsiung University of Science and Technology Kaohsiung Taiwan; ^12^ Doctoral Degree Program in Marine Biotechnology National Sun Yat‐Sen University Kaohsiung Taiwan; ^13^ Doctoral Degree Program in Marine Biotechnology, Academia Sinica Taipei Taiwan; ^14^ Joint Biobank, Office of Human Research Taipei Medical University Taipei Taiwan; ^15^ Department of Internal Medicine, Faculty of Medicine, College of Medicine Kaohsiung Medical University Kaohsiung Taiwan; ^16^ Cellular Therapy and Research Center Kaohsiung Medical University Hospital Kaohsiung Taiwan

**Keywords:** 13‐Acetoxysarcocrassolide, heat shock protein 90, leukemia, oxidative stress, topoisomerase II

## Abstract

13‐Acetoxysarcocrassolide (13‐AC) is a marine cembranoid derived from the aquaculture soft coral of *Lobophytum crassum*. The cytotoxic effect of 13‐AC against leukemia cells was previously reported but its mechanism of action is still unexplored. In the current study, we showed that 13‐AC induced apoptosis of human acute lymphoblastic leukemia Molt4 cells, as evidenced by the cleavage of PARP and caspases, phosphatidylserine externalization, as well as the disruption of mitochondrial membrane potential. The use of N‐acetylcysteine (NAC), a reactive oxygen species (ROS) scavenger, attenuated the cytotoxic effect induced by 13‐AC. Molecular docking and thermal shift assay indicated that the cytotoxic mechanism of action of 13‐AC involved the inhibition of heat shock protein 90 (Hsp 90) activity by eliciting the level of Hsp 70 and topoisomerase IIα in Molt4 cells. 13‐AC also exhibited potent antitumor activity by reducing the tumor volume (48.3%) and weight (72.5%) in the in vivo Molt4 xenograft mice model. Our findings suggested that the marine cembranoid, 13‐AC, acted as a dual inhibitor of Hsp 90 and topoisomerase IIα, exerting more potent apoptotic activity via the enhancement of ROS generation.

## 1 INTRODUCTION

Topoisomerase II (topo II) and heat shock protein 90 (Hsp 90) complex play important roles in cell mitosis and have been used as potential targets for cancer chemotherapeutic agents,^1^, ^2^ including anti‐leukemic drugs.^3^ Several topoisomerase II inhibitors including etoposide, teniposide, daunorubicin, doxorubicin, idarubicin, epirubicin, and mitoxantrone,^4^ have been commonly used in treating different leukemias such as acute myeloid leukemia (AML) and acute lymphoblastic leukemia (ALL).^5^ Currently, multi‐agent chemotherapy is the standard therapy for newly diagnosed acute leukemia cases. However, some patients may progress to relapsed or refractory disease, or develop intolerance due to drug toxicity resulting in a poor prognosis. Hsp 90 was also identified as a potential cancer target.^6^ Although no drug targeting Hsp 90 has yet been approved by the U.S. Food and Drug Administration,^7^ several clinical trials of Hsp 90 inhibitors including ganetespib (STA‐9090), are currently ongoing.^8^ There is an urgent medical need to develop new agents to treat acute leukemias. The importance of these targets in fighting leukemias encouraged scientists to search for potential inhibitors of topo II and Hsp 90 from natural sources, especially marine organisms.^9^, ^10^

**FIGURE 1:**
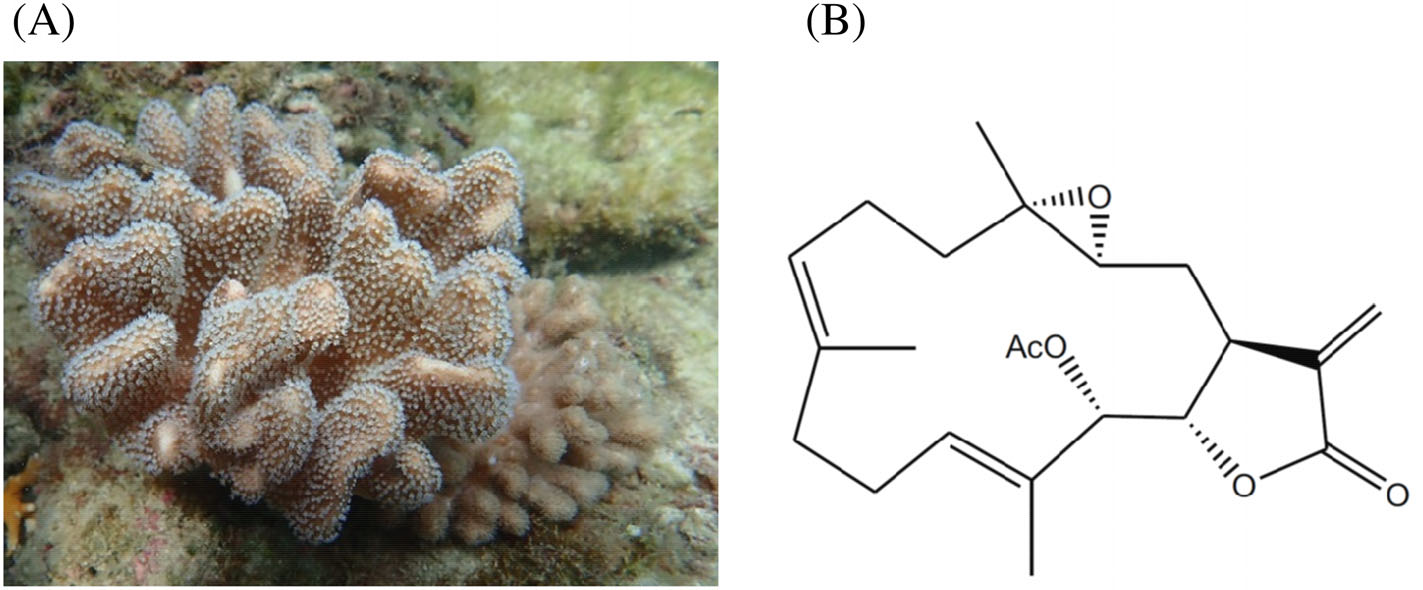
(A) Morphology of the soft coral, *Lobophytum crassum* and (B) chemical structure of 13‐AC.

Cembranolides belong to cembrane‐type diterpenoids and were organically isolated from marine organisms. They are characterized by the presence of a 14‐membered carbocyclic ring skeleton with a 5‐, 6‐, 7‐, or 8‐membered lactone ring. The cembrane derivatives from the soft coral *Lobophytum* species demonstrated several pharmacological effects including antiviral, immunostimulant, anti‐inflammatory, antitumor, and antibacterial activities.^11^ Several compounds were isolated from *Lobophytum crassum* (Figure 1A), such as lobocrassins A‐E, 14‐deoxycrassin, tetrahydrofuran cembranoids, crassumols A‐C, and 13‐acetoxysarcophytoxide (13‐AC) (Figure 1B).^2^ A modest anti‐leukemic effect was observed by lobocrassin B and culobophylins A and B. 13‐AC and other cembrane‐type diterpenoids including sarcocrassocolide M and 14‐deoxycrassin, were also active against lymphoma cells.^12^ The two cembranoids, 13‐AC, and 14‐deoxycrassin, possessing α‐methylene‐γ‐lactone or α‐methylene‐δ‐lactone functionalities, were the major components of the aquacultural soft coal as demonstrated by HPLC quantitative analysis. Among the isolated cembrane derivatives, 13‐AC, isolated from *Lobophytum crassum*, attracted attention because it exhibited potent cytotoxic activity against several human cancer cell lines including breast, colon, oral, prostate, leukemia, bladder, and gastric cancers.^13^ The feasibility of the large‐scale aquaculture and a high extraction yield of 13‐AC suggested the future potential applications of this technique to produce biologically active compounds on an industrial scale to meet the growing market demands for drug leads. Despite the potent cytotoxic activity of 13‐AC against several cancer cell lines, the precise cytotoxic mechanism of action was not explored against leukemia cells. This study aimed to evaluate the cytotoxic potential of 13‐AC along with its mechanism of action using in vitro cellular and in vivo xenograft models.

## 2 RESULTS

### 2.1 Antiproliferative and apoptotic effects of 13‐AC against leukemia and lymphoma cells

To investigate the antiproliferative activity of 13‐AC against cells from different human hematological malignancies, we examined its effect on Sup‐T1 (lymphoblastic lymphoma), U937 (lymphoma), K562 (chronic myeloid leukemia), Molt4 (acute lymphoblastic leukemia), and HEK293 (non‐hematological malignancy) cell lines using MTT cellular proliferation assay. The IC_50_ values following 13‐AC treatment for 24 and 48 h were shown in Table 1. Molt4 leukemia cells showed the highest susceptibility to 13‐AC among different cells examined, with the lowest IC_50_ level of 1.88 ± 0.39 μg/mL after 24 h and 1.63 ± 0.38 μg/mL after 48 h.

**TABLE 1:** Antiproliferative activity induced by 13‐AC against different cell lines from human hematological malignancy, including Sup‐T1 (lymphoblastic lymphoma), U937 (lymphoma), K562 (chronic myeloid leukemia), Molt4 (acute lymphoblastic leukemia), and HEK293 (non‐hematological malignancy).

Cell lines	IC_50_ (μg/mL)	
	24 h	48 h
Sup‐T1	2.97 ± 0.55	2.40 ± 1.31
U937	4.73 ± 3.33	3.15 ± 1.74
K562	4.68 ± 0.98	2.18 ± 0.69
Molt4	1.88 ± 0.39	1.63 ± 0.38
HEK293	11.75 ± 2.37	5.84 ± 0.34

*Note*: The values of IC_50_ were evaluated by calcuSyn software (Biosoft, Ferguson, MO, USA). The results are presented as the mean ± SD of three independent experiments.

**FIGURE 2:**
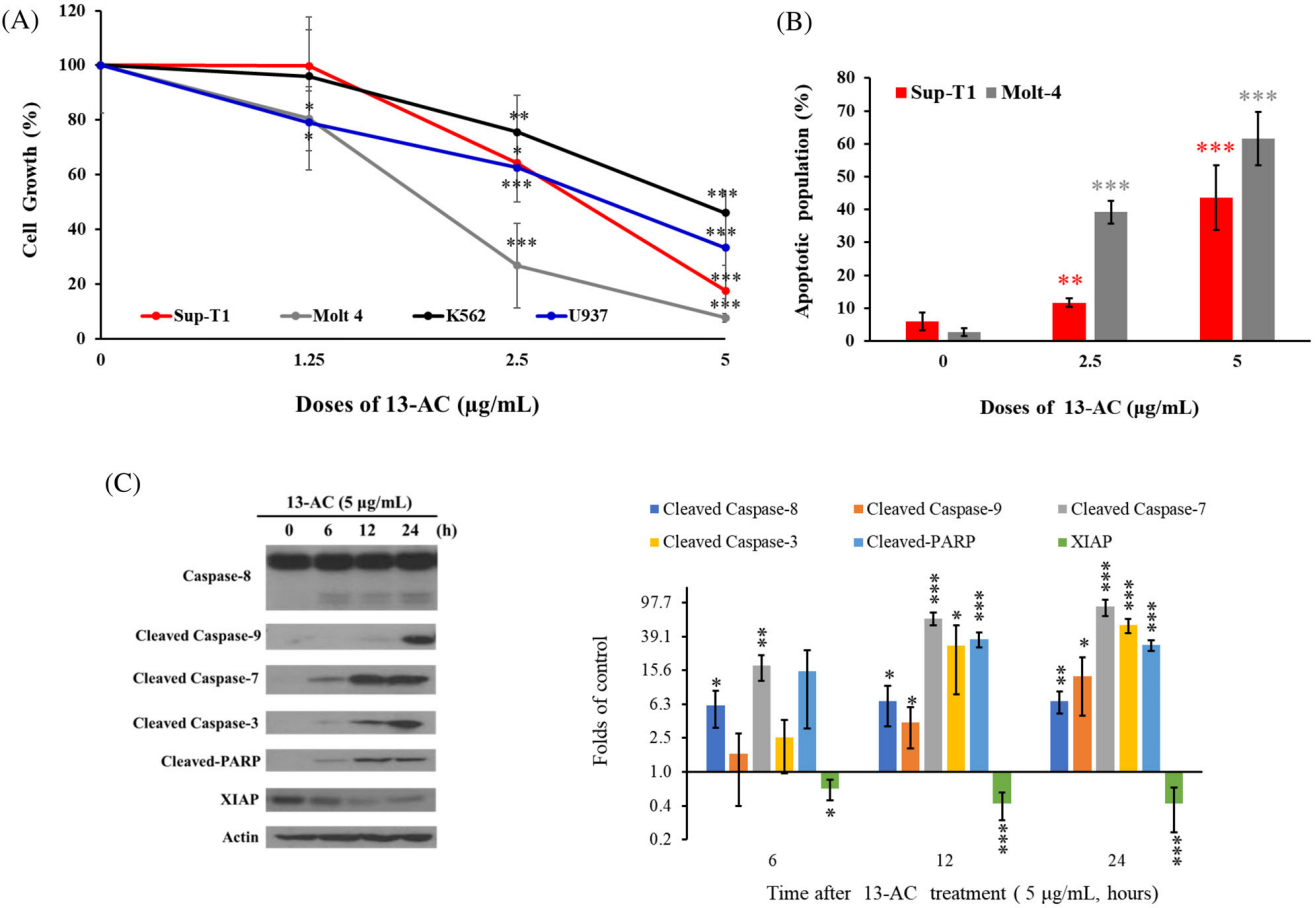
13‐AC reduced cell viability and induced apoptosis in vitro. (A) Different human leukemia and lymphoma cells were treated with several doses of 13‐AC for 24 h and the cellular growth was analyzed by MTT assay. (B) Apoptosis induction by 13‐AC in Molt4 and Sup‐T1 cells was assessed with annexin V/PI staining using flow cytometric analysis. (C) The expression of apoptosis‐related proteins in Molt4 cells treated with 13‐AC at 5 μg/mL was determined by Western blotting analysis. Actin was used as the loading control. The results are presented as means ± SD of three independent experiments (*p < 0.05; **p < 0.01; ***p < 0.001).

Next, we examined the effect of 13‐AC on growth ratio of Sup‐T1, U937, K562, and Molt4 cells. These cells were treated with increasing concentrations (1.25, 2.5, and 5 μg/mL) of 13‐AC for 24 h and the proliferation was evaluated using MTT cellular proliferation assay. 13‐AC at 5 μg/mL significantly suppressed the cellular growth in Molt4 and Sup‐T1 cells to 7.75% and 17.63%, respectively, but mildly attenuated the cellular growth to 46.15% in K562 cells and 33.38% in U937 cells (Figure 2A). To confirm the results of the MTT assay, annexin‐V/PI staining with flow cytometric analysis was conducted to examine whether the growth inhibition and the cytotoxic activity of 13‐AC were elicited by apoptosis of these cancer cells. As shown in Figure 2B, the treatment with 13‐AC at 2.5 and 5 μg/mL significantly increased the apoptotic cell population by 11.66% and 43.64% in Sup‐T1 cells, and by 39.22% and 61.60% in Molt4 cells, respectively, after 24 h of treatment. The Molt4 leukemia cell was the most susceptible to 13‐AC and was therefore selected to investigate the apoptotic mechanism of action of this cembranoid. To evaluate the expression of apoptosis‐related proteins, Molt4 cells were treated with 13‐AC at 5 μg/mL and the expression of XIAP, cleavage of caspases‐3, 7, 8, and ‐9, as well as PARP, were measured after 0, 6, 12, and 24 h by Western blotting assay. As shown in Figure 2C, 13‐AC promoted the activation of various caspases and cleavage of PARP, whereas it attenuated the expression of XIAP, a caspase inhibitor, in a time‐dependent manner. These observations suggested that 13‐AC could induce apoptosis of Molt4 leukemia cells.

### 2.2 The effect of 13‐AC on tumor growth of human leukemia Molt4 xenograft animal model

**FIGURE 3:**
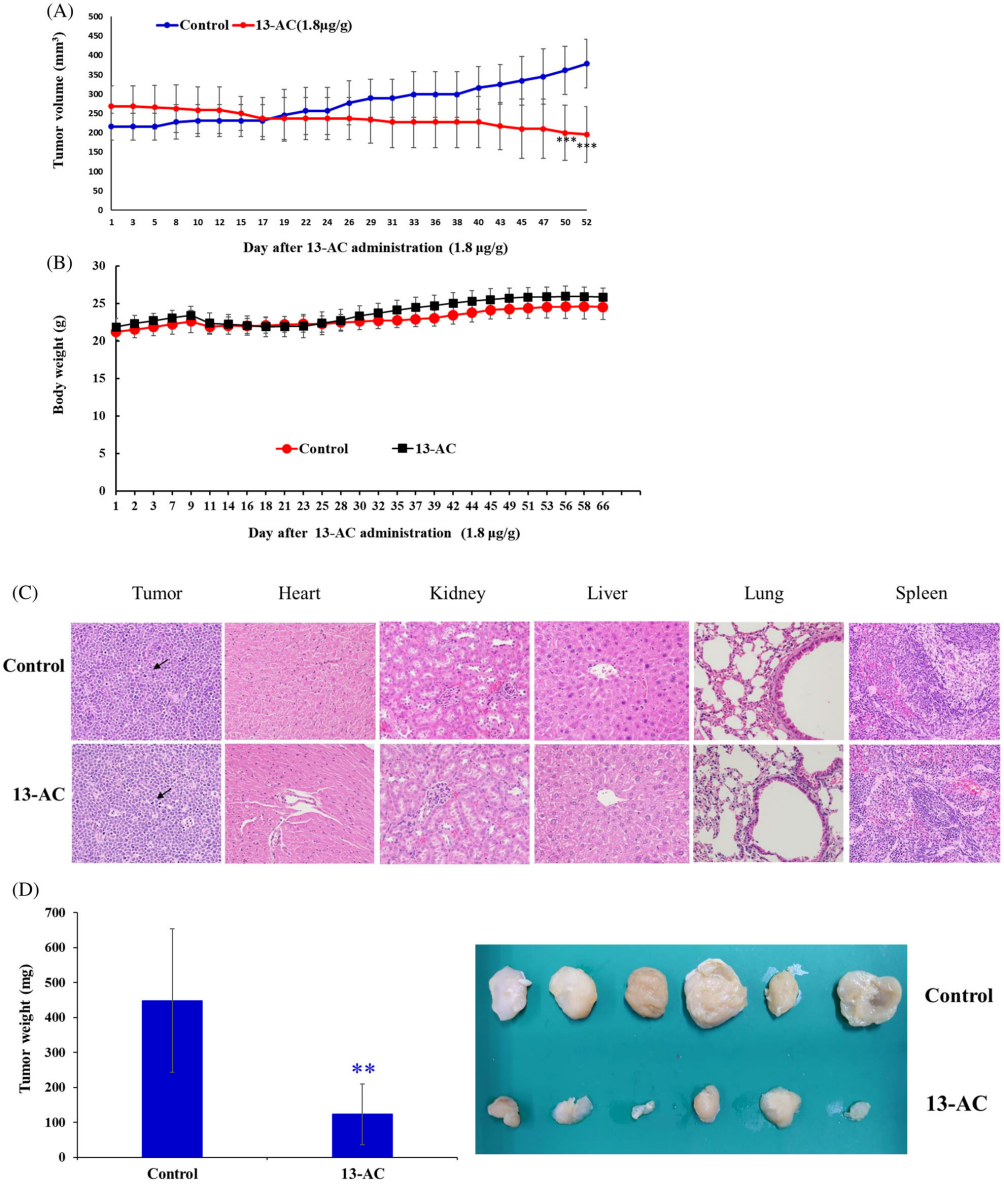
The antitumor effect induced by 13‐AC against human leukemia Molt4 cell in the xenograft model. (A) Tumor‐bearing nude mice were injected with the solvent control (DMSO, *n* = 6) and 13‐AC (1.8 μg/g, *n* = 6) by intraperitoneal injection for 52 days following the 13‐AC administration for 14 days. Tumor volumes were measured every other day. (B) The serial change of mice's body weight was measured every other day. (C) Histopathological findings from different organs after treatment with DMSO and 13‐AC in the xenograft of Molt4 cells. The tumor cells were round and showed a high nucleic/cytoplasm ratio with a high mitotic figure (arrow) in the 13‐AC treated group. No significant histopathologic changes were found in the heart, kidney, liver, lung, and spleen of the 13‐AC treated mice (H&E stain, 400×). (D) Histogram of the tumor weight from the control group and 13‐AC treated group. Representative photos of the subcutaneous tumors were collected after treatment with the solvent control (upper) group or with 13‐AC (lower) treated groups for 52 days. Data were expressed as mean ± SD. Significantly different in comparison with the control groups at *p < 0.05; **p < 0.01; ***p < 0.001.

To further explore the anticancer effect of 13‐AC on Molt4 leukemia cells in vivo, the tumor formation in the xenograft animal model was evaluated. Following the administration of 13‐AC from day 1 to day 14, the average tumor volume in the control group was 378.6 ± 62.6 mm^3^ whereas the average tumor volume in the 13‐AC‐treated group was 195.7 ± 71.8 mm^3^ on day 66 (Figure 3A). The tumor volume was reduced by 48.3% in the 13‐AC treated group in comparison with the control group. No significant body weight change was observed between the two groups, and no histopathological abnormality of the heart, kidney, liver, lung, and spleen, was observed by H&E staining between the two groups (Figures 3B,C). At the end of the experiments, the tumor tissues were isolated and weighed. The results showed a significant reduction in the average tumor tissue weight in the 13‐AC‐treated group (123.22 mg) compared with the control group (448.6 mg) (Figure 3D). A reduction in the tumor volume in the 13‐AC treated group was observed without significant changes in the mice weights. The absence of significant histopathological change in different tissues from 13‐AC treated mice suggested that 13‐AC exhibited a potent antitumor effect in vivo without significant side effects on the normal tissues.

### 2.3 Effect of 13‐AC on the homeostasis of mitochondria, cellular calcium concentration, and induction of oxidative stress

**FIGURE 4:**
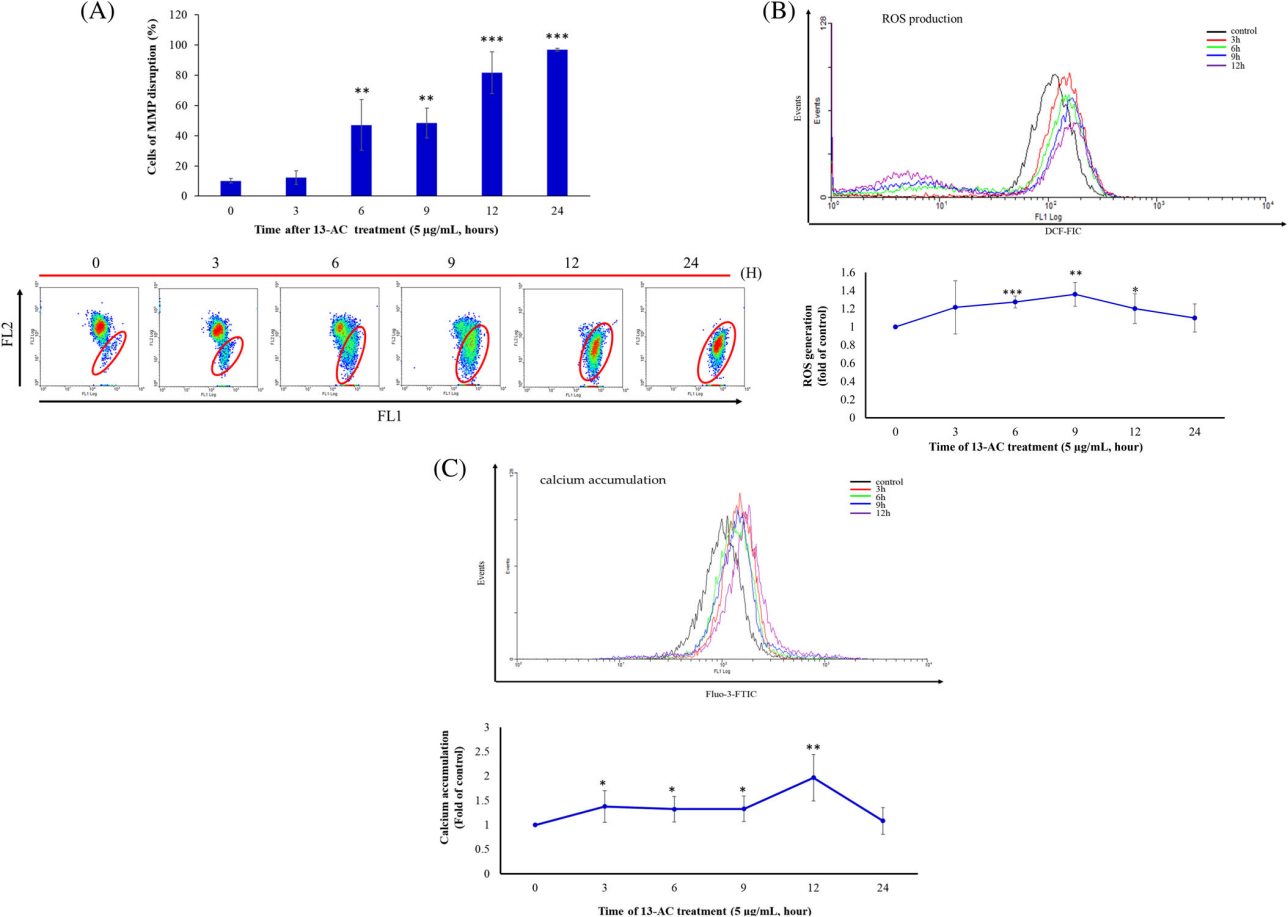
The effect of 13‐AC on the homeostasis of mitochondrial membrane potential, ROS generation, and calcium accumulation in leukemia Molt4 cells. (A) The mitochondrial membrane potential, (B) ROS generation, and (C) calcium accumulation were determined by flow cytometric assay with JC‐1, DCFH‐DA, and Fluo‐3 staining, respectively. Molt4 cells were treated with 13‐AC (5 μg/mL) at 0, 3, 6, 9, 12, and 24 h, respectively. The results are presented as means ± SD of three independent experiments (*p < 0.05; **p < 0.01; ***p < 0.001 vs. control group).

In our previous study, 13‐AC induced apoptosis in oral cancer Ca9‐22 cells via calcium release, dysfunction of mitochondrial membrane potential (MMP), and generation of reactive oxygen species (ROS).^2^ Therefore, we sought to determine whether 13‐AC exerted similar effects on leukemia Molt4 cells or not. As shown in Figure 4A, the treatment with 13‐AC (5 μg/mL) for 24 h resulted in a time‐dependent increase of the population of mitochondrial membrane potential in Molt4 cells from 10.1% to 12.3%, 47.0%, 48.5%, 81.7%, and 96.9% after 3, 6, 9, 12, and 24 h, respectively, as evaluated by JC‐1 staining. In Figure 4B, the treatment with 13‐AC (5 μg/mL) for 3, 6, 9, 12, and 24 h resulted in a 1.20‐, 1.21‐, 1.37‐, 1.41‐, and 1.13‐folds increase in the ROS levels compared with the control, as determined by carboxy‐H2DCFDA staining by flow cytometric analysis. ER stress can be induced by oxidative stress leading to mitochondrial‐dependent apoptosis.^14^ As shown in Figure 4C, an increase in cellular calcium accumulation by 1.38‐, 1.32‐, 1.33‐, 1.97‐, and 1.08‐folds was observed after the treatment with 13‐AC (5 μg/mL) for 3, 6, 9, 12, and 24 h, as determined by a fluorescent calcium indicator Fluo 3. These results indicated that 13‐AC treatment disrupted mitochondrial membrane potential, disturbed the homeostasis of calcium, and induced ROS generation leading to apoptosis in Molt4 cells.

### 2.4 13‐AC inhibited the functional activity of Hsp 90 and topoisomerase II α with a cell‐free system

**FIGURE 5:**
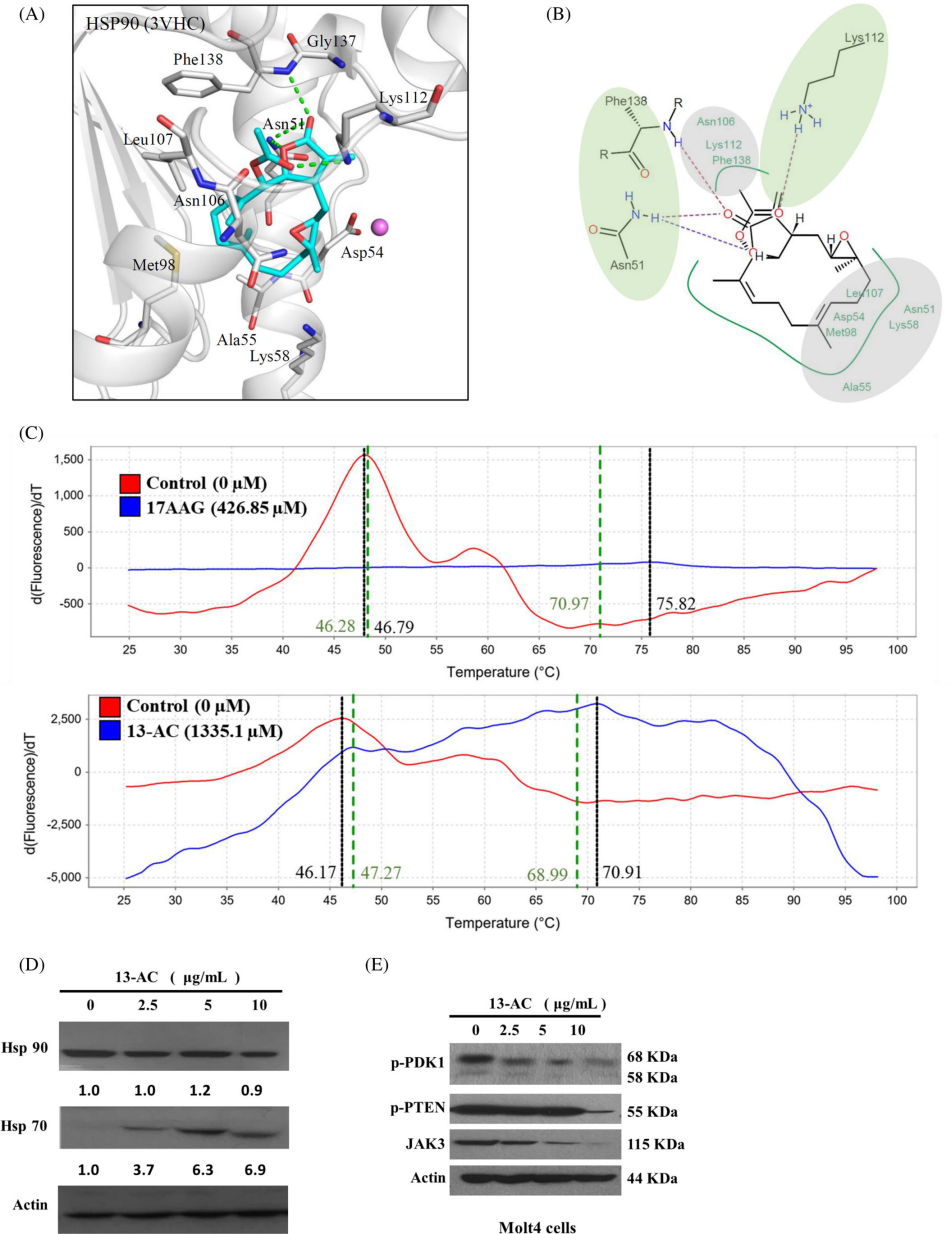
13‐AC acted as a potent inhibitor of Hsp 90. (A and B) Molecular modeling of Hsp 90 protein structure with 13‐AC as assessed by Autodock 4.2 software with Lamarckian Genetic Algorithm. (C) The melting temperature shift of 17‐AAG and 13‐AC binding to Hsp 90 protein, as examined with thermal shift assay. The effect of 13‐AC on the expression of Hsp 70 and 90 of Molt4 cells (D) and the Hsp 90 clients of Molt4 cells (E) determined by Western blotting analysis, was a dose‐dependent manner, respectively. Actin was used as the loading control. The statistical differences were denoted at *p < 0.05; **p < 0.01; ***p < 0.001 when compared with the negative control. All experiments were repeated in triplicate.

It was reported that the mitochondrial homeostasis of tumor cells is regulated by Hsp 90 chaperone network.^15^ To investigate whether the inhibition of Hsp 90 participated in the 13‐AC‐induced apoptosis of Molt4 cells, we performed a molecular docking analysis to determine the binding mode between 13‐AC and Hsp 90. As shown in Figure 5A, 13‐AC showed affinity towards Hsp 90. The tulipalin A moiety of 13‐AC created two hydrogen bonds with the residue Asn51 and one hydrogen bond with Phe138 (Figure 5A). The acetic acid moiety created a hydrogen bond with the Lys112 side chain (Figure 5B). The moiety showed hydrophobic interactions with the residues Asn106, Lys112, and Phe138. Both moieties of the 13‐AC head structure acted as hydrogen acceptors in the Hsp 90 binding site. The carbon ring structure formed hydrophobic interactions with certain residues such as Asp54, Lys58, Met98, and Leu107 (Figure 5B). 13‐AC possessed two areas that created hydrogen bonds (green) and two areas that created hydrophobic interactions (gray) (Figure 5B). According to the molecular docking experiments, 13‐AC could be docked to the N‐terminal domain of Hsp 90 with a binding energy of −8.33 kcal/mol, which was more than the first Hsp 90 N‐terminal inhibitor, 17‐AAG (−6.45 kcal/mol). 13‐AC inhibited Hsp 90 with an inhibition constant of 0.79 μM (>23.51 folds), which was more potent than 17‐AAG (18.57 μM).

To further investigate the effect of 13‐AC on the interaction of Hsp 90, a fluorescence‐based protein thermal shift assay was performed. The thermal shift assay is a method with quantitative real‐time PCR to determine the ligand (inhibitor) on the shift of the melting temperature (fluorescence intensity) of the specific protein by incrementally heating samples over‐temperature gradient (from 25 to 95°C).^16^ As shown in Figure 5C, the melting temperature (Tm) value was 70.76 ± 8.97°C in Hsp 90 protein treated with 20 μg/mL of 17‐AAG and 68.89 ± 7.44°C in Hsp 90 protein treated with 10 μg/mL of 13‐AC, respectively. The △Tm values of Hsp 90 protein treated with 17‐AAG and 13‐AC were 22.95 ± 8.97 and 22.53 ± 7.44, respectively. The thermal shift of 17‐AAG and 13‐AC binding to Hsp 90 protein with △Tm value of more than 3 suggests a potent ligand with a stronger binding force with Hsp 90. The western blotting assay showed that 13‐AC treatment (1.25, 2.5, and 5 μg/mL) did not cause changes in Hsp 90 expression compared with the solvent control. However, the expression of Hsp 70 at the same doses of 13‐AC treatment was significantly enhanced in comparison with the control in Molt4 cells (Figure 5D). According to previous studies, the upregulation of Hsp 70 expression was a biomarker of Hsp 90 inhibitor with N‐terminal inhibition.^9^, ^17^ As expected, the expression of the kinase‐related clents, p‐PTEN, p‐PDk1, and Jak 3,^18^, ^19^, ^20^ was significantly reduced at the highest dose of 13‐AC treatment in Molt4 cells (Figure 5E). Therefore, our results confirmed that 13‐AC served as an inhibitor of Hsp 90 proteins.

**FIGURE 6:**
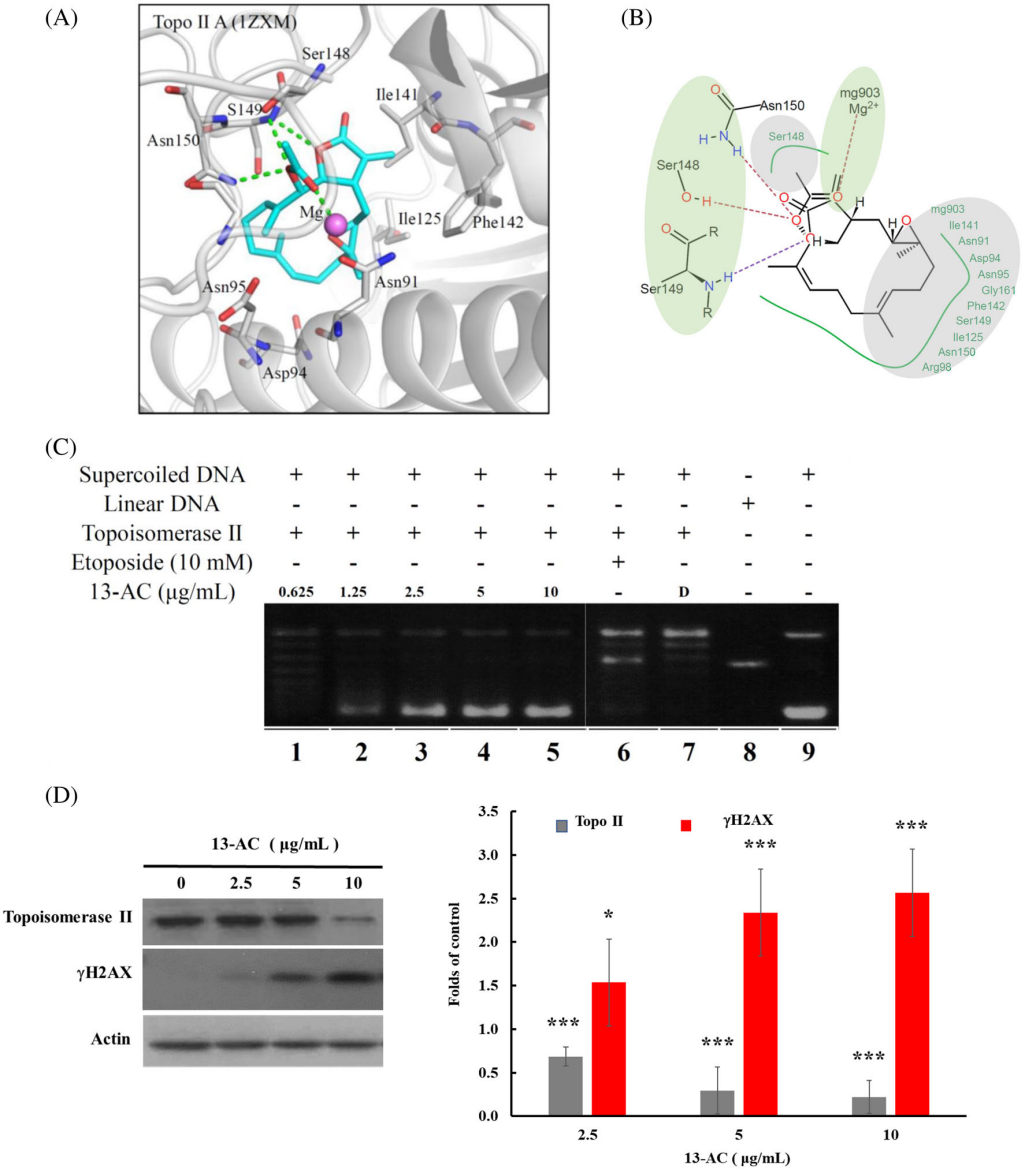
13‐AC acted as a potent inhibitor of Topoisomerase IIα. (A) 13‐AC occupies the Topo II binding site and (B) the 2D interaction map of 13‐AC in Topo II. Molecular modeling of Topo II protein with 13‐AC as assessed by Autodock 4.2 software with Lamarckian Genetic Algorithm. (C) Effect of 13‐AC on Topo II activity. Lanes 1–5: 13‐AC (0.625, 1.25, 2.5, 5, and 10 μg); Lane 6: supercoiled DNA + topo II + etoposide control (induction of DNA relaxation); Lane 7: solvent control (0 μg/mL of 13‐AC); Lane 8: linear DNA; Lane 9: supercoiled DNA. (D) The effect of 13‐AC on the expression of Topo II and γH2AX protein, determined by Western blotting analysis, was in a dose‐dependent manner. Actin was used as the loading control. The statistical differences were denoted at *p < 0.05; **p < 0.01; ***p < 0.001 when compared with the negative control. All experiments were repeated in triplicate.

Our previous study demonstrated that heteronemin induced apoptosis in prostate cancer LNcap cells with the suppression of the N‐terminal domain of Hsp 90 in an ATP‐competitive fashion along with the inhibition of Topo I and II.^10^ Clinically, Topo II was proved to be a highly promising target in cancer therapy.^21^ In the current study, we performed a molecular docking analysis to determine the molecular interactions between 13‐AC and Topo II. The docking results showed that 13‐AC interacted with the Topo II binding site. The head structure of 13‐AC, which contains a tulipalin A and the acetic acid moiety, extended into the Topo II binding site. The tulipalin A head structure created a hydrogen bond with the Topo II residue Ser149, and the acetic acid moiety formed hydrogen bonds with the residues Ser148 and Asn150 (Figure 6A). Both the tulipalin A and the acetic acid moieties of 13‐AC acted as hydrogen acceptors in Topo II (Figure 6B). The acetic acid also coordinated with the Mg^2+^ ion within Topo II (Figure 6B). The coordination to the Mg^2+^ ion was also observed with the ATP co‐crystal ligand.^22^ It was suggested that the Mg^2+^ ion was involved in DNA binding.^23^ The interactions with the Mg^2+^ ion are important for molecular recognition. The acetic acid moiety formed hydrophobic interactions with the residue Ser148. The opposite end of 13‐AC is a large carbon ring. This created hydrophobic interactions with a series of residues such as Ile125, Ile141, and Phe142 (Figure 6B). This carbon ring was also located near the periphery of the Topo II binding site. To confirm the effect of 13‐AC on Topo II activity, a cell‐free DNA cleavage system using a specific Topo II‐mediated negatively supercoiled pHOT1 plasmid DNA was applied.^10^ As shown in Figure 6C, the use of 13‐AC at a concentration of more than 2.5 μg/mL significantly attenuated the DNA relaxation compared with the solvent control, DMSO, and resulted in the formation of supercoiled DNA products in the presence of Topo IIα (Lanes 3–5). A linear DNA product was obtained with the use of Topo II poison, etoposide (Lane 6). We also examined the effect of 13‐AC on the expression of Topo II protein by Western blotting assay. As shown in Figures 6D, 13‐AC treatment suppressed the expression of Topo II protein and promoted the expression of λH2AX (a biomarker of DNA double‐strand breaks) at the concentrations 13‐AC of 5 and 10 μg/mL, suggesting that 13‐AC treatment could inhibit the catalytic effect of Topo II and induce DNA double‐strand breaks.

### 2.5 13‐AC cytotoxic effect is mediated through oxidative stress and Hsp 90 inhibition

**FIGURE 7:**
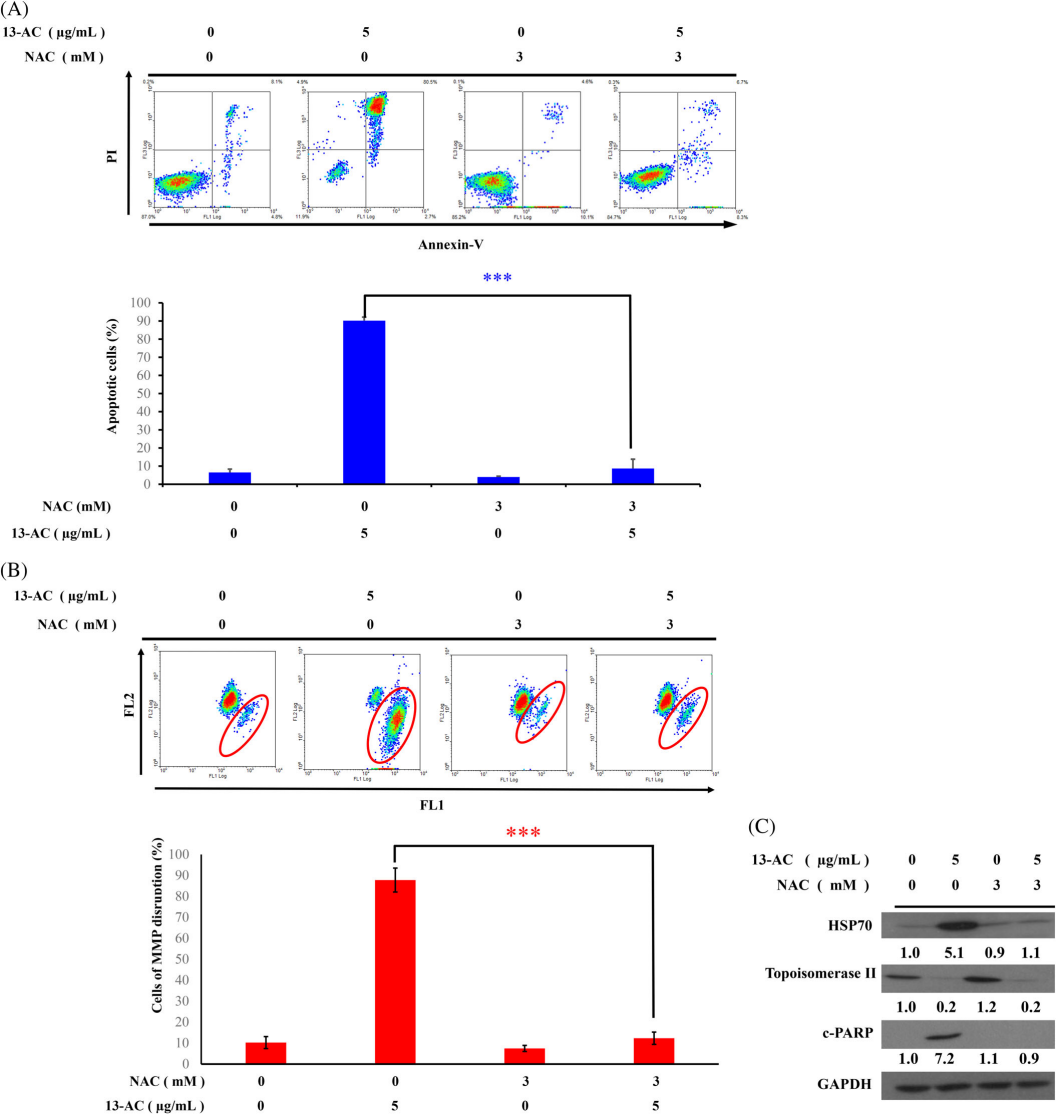
The apoptotic effect of 13‐AC involved the induction of oxidative stress in Molt4 cells. (A) The apoptosis and (B) disruption of MMP populations were examined with annexin‐V/PI and JC‐1 staining, respectively. The cells were pretreated with 3 mM of NAC for 2 h, then treated with 5 μg/mL of 13‐AC for a further 24 h; (C) Effect of NAC pretreatment on the expression of *c*‐PARP, Hsp 70, and Topo IIα alone or in a combination of 13‐AC treatment were examined by the Western blotting assay. GAPDH was used as the loading control. The results are presented as means ± SD of three independent experiments (*p < 0.05; **p < 0.01; ***p < 0.001) when compared with the group treated with 13‐AC.

Oxidative stress plays an important role in cancer therapy. We investigated whether the 13‐AC‐induced cell apoptosis is mediated through ROS generation. In our previous study, N‐acetyl‐L‐cysteine (NAC), a ROS scavenger, reduced the 13‐AC induced cell apoptosis and MMP in Ca9‐22 cells.^2^ To identify whether ROS plays a similar role in 13‐AC induced apoptosis in Molt4 cells, a flow cytometric assay was performed to determine the population of apoptotic cells after NAC pretreatment. The pretreatment of Molt4 cells with NAC suppressed 13‐AC‐induced apoptosis (Figure 7A). The annexin V/PI staining assay suggested that 13‐AC induced apoptosis by ROS generation and was blocked by NAC. Since 13‐AC acted as a dual inhibitor of Hsp 90 and Topo IIα in the molecular docking assay, Molt4 cells were either subjected to 13‐AC or 13‐AC and NAC pretreatment. We observed a decrease of Topo II and an increase of Hsp 70 in response to 13‐AC treatment alone. However, 13‐AC in combination with NAC pretreatment significantly attenuated the expression of Hsp 70 and restored the suppressed expression of Topo II (Figure 7B). The present study suggested that the cytotoxic activity of 13‐AC is mediated through the inhibition of Hsp 90 function and the induction of oxidative stress in leukemia cells.

## 3 DISCUSSION

Multiagent chemotherapy remains the standard first‐line therapy for newly diagnosed ALL. The overall survival rate is largely influenced by age, with a 5‐year survival rate in 80% of patients under the age of 50 but only 35% in patients above 50 years old.^24^ The poor prognosis in older patients is likely due to the adverse risks of disease biology, and higher treatment‐related complications due to underlying comorbidities. Therefore, there is a medical need to develop novel agents with different mechanisms of action to tailor treatment, to fit the disease and patient profile.

Corals are a rich reservoir of biologically active secondary metabolites such as cembranolides that can act as drug leads for the development of cancer therapeutics.^12^ These marine cembranolides exhibited a potent cytotoxic effect against several cancer cells such as AGS (human gastric adenocarcinoma), BTFC (bladder transitional cancer), MCF‐7 and T47D (breast cancer), Hela (cervical cancer), DLD‐1, HCT116, and Lovo (colon cancer), Ca9‐22 and Cal‐27 (oral cancer), DU145 and LNcap (prostate cancer) cells.^2^ 13‐AC is a marine natural cembranoid with a unique chemical structure showing a specific functionality of α‐methylene‐γ‐lactone. It was isolated from the crude extracts of aquaculture *Lobophytum crassum* and *Sarcophytum* sp. It showed several pharmacological effects including antiviral, immunostimulatory, anti‐inflammatory, antitumor, and antibacterial activities.^11^, ^25^, ^26^, ^27^ Despite the cytotoxic activity of 13‐AC against several cancer cells, its antileukemic effect was not yet explored. This study demonstrated the anti‐leukemic mechanism of action of 13‐AC using in vitro and in vivo models for the first time. 13‐AC treatment depolarized the mitochondrial membrane leading to the opening of the mitochondrial permeability transition pore, as demonstrated by JC‐1 staining (Figure 4A). It also induced MMP disruption and apoptosis through a combination of oxidative and calcium accumulation, as demonstrated by flow cytometric results (Figure 4B,C). The results were consistent with our previous study on a different cancer cell line.^2^ The in vivo experiments showed that 13‐AC inhibited the tumor growth of Molt4 cells in a xenograft animal model and did not affect the body weight of the treated mice. The blood biochemical values were in the permissible range, suggesting no adverse effect on the liver and kidney functions in mice treated with 13‐AC. The cell‐free system and computational modeling using structure–function analysis further supported that 13‐AC can act as a potential dual topoisomerase catalytic and Hsp 90 inhibitor (Figures 5 and 6).

Based on the docking results, we found that 13‐AC interacted with the binding sites of Hsp 90. The head structure of 13‐AC contains tulipalin A and acetic acid moieties. This configuration allowed the two areas of the head structure to form hydrogen bonds and one area to form hydrophobic interaction. For instance, the acetic acid moiety formed a hydrogen bond with Hsp 90 residue Lys112. The second hydrogen bond was formed by the tulipalin A region and Phe138 residue of Hsp 90 (Figure 5A,B). The head region acted as a point for the hydrophobic interactions of Hsp 90. The head of 13‐AC is connected to a large carbon ring forming hydrophobic interactions with several residues in Hsp 90. This section was aligned within the active site periphery of Hsp 90. The docking results suggested that the 13‐AC head structure anchored the compound to the target protein binding sites. We found that the structure of 13‐AC shared similar interaction preferences with Hsp 90 allowing the compound to infiltrate the binding sites of Hsp 90. These observations suggested that 13‐AC is a potent Hsp 90 inhibitor.

13‐AC also interacted with the binding sites of both Topo II and Hsp 90. There were similarities between the interactions observed in both target protein binding sites. The head structure of 13‐AC contains a tulipalin A and acetic acid moiety. This allowed the two areas of the head structure to form hydrogen bonds and one area to form hydrophobic interactions. For instance, the acetic acid moiety forms a hydrogen bond with Topo II residue Ser148 and Hsp90 residue Lys112. The second hydrogen bond area was formed by the tulipalin A region and can be observed with Topo II residue Ser149 and Hsp 90 residue Phe138 (Figure 5A,B). The head region was also a point for hydrophobic interactions for both Topo II and Hsp 90. The head of compound 13‐AC is connected to a large carbon ring that formed hydrophobic interactions with several residues in Topo II and Hsp 90. This section was found to be aligned within the active site periphery of both Topo II and Hsp 90. This suggested that the 13‐AC head structure anchors the compound in both target protein binding sites. The molecular docking analysis showed that the structure of 13‐AC shares similar interaction preferences. Recent studies indicated that the N‐terminal domain of Hsp 90 possesses an ATP binding similar to the bacterial Topo II and DNA gyrase.^28^ This similarity might provide a rational explanation for the compound infiltration of the binding sites of both Topo II and Hsp 90. These observations suggested that 13‐AC is a dual Topo II and Hsp 90 inhibitor.

Hsp 90 is responsible for the folding of selected oncogenic proteins and plays an essential role in cancer progression, tumor growth, adhesion, metastasis, invasion, and angiogenesis. The suppression of the Hsp 90 protein folding machinery results in a combinatorial attack on numerous oncogenic kinase pathways, such as the clients of Hsp 90, ALK, and Akt.^29^ Moreover, caspase activation occurs with the cleavage of ALK and PLCλ1, receptor tyrosine kinases during apoptosis.^30^ Given the characteristic cancer chaperone of Hsp 90, it can act as a hub for many neoplastic signaling pathways involved in the regulation of cell survival and the growth inhibitory activities in cancer cells.^31^ The activity of Hsp 90 inhibitors might be dependent on the balance of pro‐apoptotic and pro‐survival regulatory proteins.^32^ The N‐terminal inhibitors of Hsp90 failed in the clinic as a single therapy treatment partially because they induce a heat shock response.^33^ Moreover, Hsp 90 inhibitors, AT‐533, inhibited tumor growth and angiogenesis via the blockage of HIF‐1 binding and degradation of HIF‐1 in breast cancer.^34^ The overexpression of Hsps is heavily dependent in many cancer cells on the upregulation of transcript factor HSF‐1 which is the multifaceted crucial regulator of the heat shock response.^35^ It is worth to note that Hsp 90 inhibitors competed at ATP‐binding site with inhibition of the ATPase activity and ATP‐ADP exchange to induce the degradation of clients via the proteasome mechanism and recruited the ligation of ubiquitin and Hsp90. Mitochondria serve as an important bioenergy hub to regulate the ATP production, ROS generation and apoptotic induction.^36^ In this study, the generation of ROS was increased in Molt4 cells after 13‐AC treatment. We found that the pretreatment of NAC, a ROS scavenger, attenuated MMP disruption and apoptosis induction (Figure 7A,B), as well as increased Hsp 70 expression (Figure 7C) induced by 13‐AC. From these results, we speculated that the cytotoxicity of 13‐AC, a specific Hsp 90 inhibitor, suppressed the growth of Molt4 cells by the induction of mitochondria‐dependent apoptosis via ROS generation.

## 4 MATERIALS AND METHODS

### 4.1 Cell culture and chemicals

The American Type Culture Collection (ATCC, Manassas, VA, USA) was the source of Melt4 cell lines. RPMI 1640 medium was the selected medium and the cells were maintained at 37 °C in 5% CO_2_. The medium contained fetal calf (10%) serum, glutamine (2 mM), and antibiotics (100 μg/mL of streptomycin and 100 units/mL of penicillin). From Strong Biotech Corporation (Taipei, Taiwan) we obtained Annexin V‐FITC/PI (propidium iodide) stain. Sigma‐Aldrich (St. Louis, MO, USA) was the source of 3‐(4,5‐dimethylthiazol‐2‐yl)‐2,5‐diphenyl‐tetrazolium bromide (MTT) and dimethyl sulfoxide (DMSO) and all other chemicals. Molecular Probes and Invitrogen technologies (Carlsbad, CA, USA) were the sources of the carboxy derivative of fluorescein (carboxy‐H_2_DCFDA), Fluo‐3, and JC‐1 cationic dye. Amersham Life Sciences (Amersham, UK) was the source of ECL Western blotting detection kits and Hybond ECL transfer membrane.

### 4.2 Annexin V/PI apoptosis assay

Annexin V‐FITC kit was used for the externalization of PS and membrane integrity. In a 35 mm dish, 10^6^ cells were grown. Before harvesting, the cells were labeled with annexin V‐FITC (10 μg/mL) and PI (20 μg/mL). All dishes were washed with the binding buffer after labeling and were harvested. Before the analysis by FACS‐Calibur (Becton‐Dickinson, San Jose, CA, USA) and CellQuest software, the cells were resuspended and collected with the binding buffer at a concentration of 2 × 10^5^ cells/mL.^37^

### 4.3 Determination of ROS generation, calcium accumulation, and MMP disruption

In these assays, we followed previously described protocols.^38^ JC‐1 cationic dye (5 μg/mL) was used to detect MMP disruption. A fluorescent calcium indicator (Fluo 3, 5 mM) was used to detect calcium accumulation. A carboxy derivative of fluorescein (carboxy‐H_2_DCFDA, 1.0 mM) was used to detect ROS generation. The fluorescent dye labeled the treated cells for 30 min. The cells were washed after labeling and resuspended in PBS with a concentration of 1 × 10^6^ cells/mL before analysis by flow cytometry.

### 4.4 Western blotting analysis

Cells were collected and lysed with RIPA lysis buffer, that contained 0.1% sodium dodecyl sulfate (SDS), 1% nonidet P‐40, 1 mM sodium orthovanadate, 0.5% sodium deoxycholate, 100 μg/mL phenylmethylsulfonyl fluoride, and 30 μg/mL aprotinin (Sigma Aldrich was the source of all chemicals). A BCA protein assay kit (Pierce) was used to determine the protein concentration. 7.5%, 10%, or 12% of SDS‐gel electrophoresis was used to separate equal amounts of proteins and then transferred to the PVDF membrane. Immunoblotting using specific antibodies was used to monitor the levels of the proteins. Antibodies of the cleaved caspases 3, 7, 8, and ‐9, Jak3, p‐PDK1^(ser241)^, were purchased from Cell Signaling Technologies (Beverly, MA, USA). Santa Cruz Biotechnology (Santa Cruz, CA, USA) was the source of antibodies for cleaved‐PARP, XIAP, p‐PTEN^(Ser 380)^, and topoisomerase IIα. Hsp 70 and 90 antibodies were purchased from Enzo life sciences (Taipei, Taiwan). GE Healthcare UK (Rockford, IL, USA) was the source of ECLTM anti‐mouse and rabbit IgG Horseradish peroxidase‐linked whole antibodies. An enhanced chemiluminescence kit (ECL, Amersham Life Sciences, UK) was used to examine the changes in these proteins.

### 4.5 Assay of topoisomerase II catalytic inhibitors and poisons

This assay was performed as previously described. Standard relaxation reaction mixtures (20 μL), 1 mM ATP, 0.3 μg of pHOT1 plasmid DNA, two units of human topoisomerase II (Topogen, Columbus, OH, USA) were mixed well with the indicated concentrations of etoposide and 13‐AC and were then incubated at 37°C for 30 min. Ten percent of SDS (2 μL) was added to terminate the reaction. Subsequently, 2.5 μL of proteinase K (50 μg/mL) was added to digest the bound protein (incubated at 37°C for 15 min). The DNA products were examined with electrophoresis via vertical 2% agarose gels at 2 V/cm in 0.5X TAE buffer. The agarose gels were stained with ethidium bromide and photographed using an Eagle Eye II system (Stratagene, La Jolla, CA, USA).

### 4.6 13‐AC treatment of human leukemia Molt4 cells in xenograft animal model

Previous literature was followed in the preparation of the nude mice xenografts model with some modifications. National Laboratory Animal and Research Center (Taipei, ROC) was the source of 6‐week‐old male immunodeficient athymic mice. The Animal Care and Treatment Committee of Kaohsiung Medical University (IACUC Permit Number 108152) approved this study. The Guide for the Care and Use of Laboratory Animals of the National Institutes of Health was followed in all experiments. All efforts were made to minimize animal stress/distress. First, mice were divided into two groups, DMSO (control) or 13‐AC (1.8 μg/g) treatment by intraperitoneal injection that was administrated every other day for 14 days. Molt4 cells (1 × 10^6^) were resuspended in 0.2 mL PBS, injected into the right flank of each mouse by *s.c*., and tumor growth was monitored every day. DMSO and 13‐AC administration was continued for additional 52 days. The animals were sacrificed by carbon dioxide. Tumor size was measured three times a week using calipers and the tumor volumes were calculated according to the standard formula: width^2^ × length/2.

### 4.7 Molecular docking assay

The docking analysis was performed by LeadIT, a molecular docking software.^39^ The Protein Data Bank was used to download the Topo II (PDB ID: 1ZXM) and Hsp 90 (PDB ID: 3VHC) crystal structures.^40^ Protein structures were prepared by removing water molecules and the co‐crystal ligand using the molecular docking software LeadIT. The binding sites were determined as a radius of 10 Å from the co‐crystallized ligands. The docking strategy used an enthalpy and entropy approach. The maximum number of solutions per iteration and fragmentation was set at 300. All other parameters were used at the default settings.

### 4.8 Protein thermal shift assay

According to the manufacturer's protocol (Thermo Fisher Scientific Baltics, Vilnius, Lithuania), HSP 90 (Enzo life sciences, Taipei, Taiwan) was recommended to be used as the protein (1 μg/μL/reaction) as well as 1 μL of 17AAG (20 μg) and 13‐AC (10 μg) was used as the ligand. A thermal shift buffer (5 μL), 2.5 μL of 8X Sypro Orange fluorescent dye, and 10.5 μL of water (the final volumes of 20 μL) were added to each well using a Multidrop Combi Reagent Dispenser (Thermo Fisher Scientific, Waltham, MA, USA). The plate was then sealed with MicroAmp Optical Adhesive Film (Applied Biosystems, Foster City, CA, USA) and the spun‐down reaction was mixed at the bottom of the plate. The plate was heated from 25 to 99°C at a heating rate of 1 °C/min and was measured with the ROX dye channel (Ex/Em: 490 nm/530 nm) on the Step One Plus Real‐Time PCR instrument (Applied Biosystems, Foster City, CA, USA). The melting temperatures (Tm) and thermal profiles were conducted using Protein Thermal Shift Software (version 1.3, Applied Biosystems, Foster City, CA, USA).

### 4.9 Statistics

The results were expressed as mean ± standard deviation (SD). Each experiment was performed using an unpaired Student's *t*‐test. A *p‐*value of less than 0.05 was considered to be statistically significant.

## 5 CONCLUSIONS

We found that the treatment of 13‐acetoxysarcocrassolide, a marine cembranoid, induced mitochondrial dysfunction and oxidative stress leading to apoptosis of Molt4 cells. The in vivo experiments showed that 13‐AC prevented the tumor growth of Molt4 xenograft and did not affect the body weight of the treated mice. The blood biochemical values were in the permissible range, suggesting no adverse effects on the liver and kidney functions in mice treated with 13‐AC. The thermal shift assay, cell‐free system, and computational modeling using structure–function analysis further evidenced that 13‐AC can act as a potential dual topoisomerase catalytic and Hsp 90 inhibitor. Our study provided further insights into the pro‐apoptotic mechanism of the isolated marine derivative, and their clinical potential as novel dual Hsp 90 and topoisomerase II catalytic inhibitors to target leukemia and lymphoma. Based on our results, this marine cembranoid with the unique moiety functionality of α‐methylene‐γ‐lactone represents an interesting molecular architecture that binds preferentially to oncogenic proteins, such as Hsp 90 and topoisomerase IIα.

## CONFLICT OF INTEREST STATEMENT

All authors declare no conflict of interest.
